# Synthesis and Evaluation of the Effects of Nanoparticles of Atorvastatin and Atorvastatin on Cuprizone-Induced Demyelination: Modulation of the Nrf2/NF-kB Signaling Pathway

**DOI:** 10.5812/ijpr-170319

**Published:** 2026-07-13

**Authors:** Samira Shirooie, Niloofar Heidarizade, Tayebeh Noori, Antoni Sureda, Amin Iranpanah, Sina Alibeygi

**Affiliations:** 1 Pharmaceutical Sciences Research Center, Health Institute, Kermanshah University of Medical Sciences, Kermanshah, Iran; 2 Student Research Committee, Faculty of Pharmacy, Kermanshah University of Medical Sciences, Kermanshah, Iran; 3 CIBER Fisiopatología de la Obesidad y Nutrición (CIBEROBN), Instituto de Salud Carlos III (ISCIII), 28029, Madrid, Spain; 4 Research Group on Community Nutrition and Oxidative Stress (NUCOX) and Health Research Institute of Balearic Islands (IdISBa), University of Balearic Islands, Palma de Mallorca E-07122, Balearic Islands, Spain; 5 Student Research Committee, School of Medicine, Kermanshah University of Medical Sciences, Kermanshah, Iran

**Keywords:** Demyelination, Cuprizone, Atorvastatin, Nrf2, NF-κB P65

## Abstract

**Background:**

Multiple sclerosis (MS) is a chronic inflammatory disease of the central nervous system (CNS) characterized by damage to the myelin sheath and, over time, by progressive neurological disability. Statins have attracted attention in MS research owing to their anti-inflammatory and neuroprotective effects.

**Objectives:**

This study assessed whether atorvastatin could ameliorate cuprizone-induced behavioral and histopathological changes in the brains of male C57BL/6 mice.

**Methods:**

All groups of mice, except the control group, which received a normal diet, were fed 0.2% cuprizone (CPZ) in their daily diet for 6 weeks to induce demyelination. The treatment groups received atorvastatin (1, 2, or 4 mg/kg/day, i.p.) or nano-atorvastatin (2 mg/kg/day, i.p.) during the final 2 weeks of the study. At the end of the study, behavioral tests were conducted, and immunofluorescence assessment of NF-kB p65 and Nrf2 in the corpus callosum was conducted.

**Results:**

CPZ caused progressive weight loss by the end of the study compared with the control group; this effect was reversed by treatment with atorvastatin and nano-atorvastatin. All behavioral tests showed reduced motor coordination in the CPZ group (P < 0.001) compared with the control group. Administration of atorvastatin and nano-atorvastatin during the last 2 weeks reversed these motor deficits. Histopathological examination showed significant demyelination in the CPZ group, which was reversed by atorvastatin injections. Furthermore, CPZ significantly decreased Nrf2 levels (P < 0.001) and increased NF-kB p65 levels (P < 0.001) in the corpus callosum; these changes were stabilized in the atorvastatin and nano-atorvastatin groups.

**Conclusions:**

Atorvastatin may attenuate CPZ-induced toxicity by reducing demyelination and modulating the NF-kB p65 and Nrf2 signaling pathways.

## 1. Background

Multiple sclerosis (MS) is a chronic inflammatory disease of the central nervous system (CNS) that damages the myelin sheath and, over time, leads to progressive neurological disability (1, 2). The condition can involve a wide range of motor, sensory, and cognitive disturbances, often profoundly affecting daily life (3). Pathologically, MS is characterized by immune-mediated myelin destruction, axonal injury, and reactive gliosis (4, 5).

It is estimated that more than 2.3 million people worldwide are living with MS, with women affected more frequently than men (6). The precise cause remains unclear, but a combination of genetic predisposition, environmental exposures, and immune system dysregulation is thought to drive disease onset and progression (7, 8). Inflammation and oxidative stress are recognized as key contributors to tissue injury in MS, with elevated reactive oxygen species (ROS) levels and impaired antioxidant responses frequently reported in patients (9, 10).

Treatment for MS typically falls into two main categories: disease-modifying therapies (DMTs), which aim to slow disease progression, and symptomatic treatments, which address specific clinical manifestations (11). DMTs can reduce relapse rates and CNS inflammation, but their benefits are often limited by adverse effects, incomplete efficacy, and poor long-term adherence (12, 13). Corticosteroids remain a mainstay for managing acute relapses, yet prolonged use is linked to complications such as weight gain, mood changes, and bone loss (14). These limitations underscore the need for new therapeutic options that can control inflammation and protect neural tissue, ideally with fewer systemic drawbacks (15).

Statins, best known for lowering cholesterol, have attracted attention in MS because of their anti-inflammatory and neuroprotective effects (16-18). Experimental studies of atorvastatin, simvastatin, and rosuvastatin have shown benefits such as reduced relapse frequency, improved remyelination, and modulation of immune activity in autoimmune and toxin-induced models of demyelination (19-21). However, results from human studies have been mixed, and higher doses carry risks, including myopathy, liver toxicity, and metabolic adverse effects (22, 23). This has led to growing interest in strategies to improve the therapeutic performance of statins while minimizing systemic adverse effects. Among these approaches, nanotechnology-based delivery systems have been proposed to enhance drug bioavailability, tissue distribution, and therapeutic efficiency (24).

Nanoparticle-based drug delivery systems are a promising strategy for improving the pharmacokinetic properties of therapeutic agents, particularly those with poor aqueous solubility. Liposomal formulations can enhance drug stability, modify tissue distribution, and provide sustained-release profiles (18, 25). Liposomal formulations have been widely investigated as drug delivery systems because they can improve the solubility, stability, and pharmacokinetic behavior of therapeutic agents (26, 27). Previous studies have demonstrated the neuroprotective and anti-inflammatory properties of atorvastatin in demyelinating and neuroinflammatory conditions (28). For atorvastatin, nano-formulation may represent a strategy to optimize drug delivery and modify tissue distribution. However, the extent to which these properties translate into improved efficacy in experimental demyelinating disorders remains incompletely understood and warrants further investigation.

Although previous studies have suggested potential anti-inflammatory and neuroprotective effects of statins in demyelinating disorders, the efficacy of atorvastatin has not been consistently demonstrated across experimental and clinical studies. Moreover, little is known about whether the nano-formulation of atorvastatin could influence its therapeutic performance in demyelinating conditions. Therefore, further investigation is required to evaluate both the effects of atorvastatin itself and the potential contribution of nano-based delivery systems in experimental models of MS, as well as their potential influence on NF-kB/Nrf2 signaling.

From a mechanistic standpoint, the NF-kB pathway is a central driver of inflammation in MS, promoting cytokine production and glial-cell activation (29, 30). In contrast, the Nrf2 pathway acts as a defense system against oxidative damage by regulating antioxidant gene expression (31). When NF-kB is overactivated and Nrf2 signaling is impaired, a damaging cycle of neuroinflammation and oxidative stress can occur (28, 32, 33). Strategies that can simultaneously suppress NF-kB and enhance Nrf2 activity could therefore offer meaningful neuroprotection (34, 35).

## 2. Objectives

In the present study, we evaluated the effects of atorvastatin and a nano-atorvastatin formulation in a cuprizone (CPZ)-induced mouse model of MS. We assessed behavioral performance and conducted histological analyses to evaluate motor coordination, demyelination, and neuroinflammation, with particular attention to changes in NF-κB and Nrf2 signaling (36).

## 3. Methods

### 3.1. Materials

Atorvastatin, lecithin, and chloroform were purchased from Sigma-Aldrich, and CPZ was obtained from Merck KGaA, Germany. The following antibodies were used for immunofluorescence analysis: mouse monoclonal antibody NF-kB p65 (sc-8008), rabbit monoclonal antibody Nrf2 (phospho S40) (ab76026), and m-IgGk BP-HRP (sc-516102) as the secondary antibody.

### 3.2. Preparation of Nano-Atorvastatin

Nano-atorvastatin was prepared using a modified thin-film hydration and sonication method. Briefly, 72 mg of lecithin and 24 mg of atorvastatin calcium were dissolved in 2.4 mL of chloroform to obtain a homogeneous lipid–drug mixture. The organic solvent was then removed by rotary evaporation to form a uniform thin film of lecithin and atorvastatin. The film was subsequently hydrated with 100 mL of distilled water. To achieve the desired nanoparticle size, the suspension underwent a multistep sonication process: 1 minute of bath sonication, followed by 4 to 5 minutes of probe sonication (Sonopuls Ultrasonic Homogenizer HD 2070, Berlin), and a final 20 minutes of bath sonication. Throughout the procedure, the temperature was maintained at a low level to prevent thermal degradation of atorvastatin. The resulting nano-atorvastatin suspension was transferred into light-protected Falcon tubes and stored at 4 °C until further use (37).

### 3.3. Characterization of Nano-Atorvastatin

The particle size distribution and polydispersity index (PDI) of nano-atorvastatin were measured using a dynamic light scattering (DLS) instrument (Zetasizer, Malvern Instruments Ltd, UK). Measurements were performed in disposable sizing cuvettes using phosphate buffer as the dispersant at 25 °C. The zeta potential of the nanoparticles was determined using the same instrument equipped with a clear disposable zeta cell, using distilled water as the dispersant. All measurements were conducted in triplicate.

Fourier transform infrared spectroscopy (FTIR) analysis was performed using a spectrophotometer (Shimadzu IR2000, Japan) to investigate potential interactions between atorvastatin and formulation excipients. Samples of pure atorvastatin, lecithin, and freeze-dried nano-atorvastatin were scanned over the wavenumber range of 500 to 4000 cm^-1^ at a scan rate of 8/cm for 100 scans, and the resulting spectra were analyzed for characteristic peaks.

In vitro drug release was assessed using the dialysis bag diffusion method. A dialysis membrane (molecular weight cut-off, 12 kDa) containing a known volume of freshly prepared nano-atorvastatin suspension was immersed in 50 mL of phosphate-buffered saline (PBS, pH 7.4) maintained at 37 ± 0.5 °C with constant stirring at 500 rpm. At predetermined time intervals (0.5, 1, 2, 4, 6, 8, 12, 24, 36, and 48 hours), 2 mL of release medium was withdrawn and replaced with an equal volume of fresh PBS. The concentration of released atorvastatin was determined spectrophotometrically at 245 nm using a UV-vis spectrophotometer.

### 3.4. Determination of Drug Encapsulation Efficiency and Drug Loading

Drug loading (DL) and drug encapsulation efficiency (DEE) of atorvastatin-loaded nanoparticles (Atorvastatin-NPs) were determined using a UV-vis spectrophotometer. Quantification of atorvastatin content in nanoparticles was performed based on a calibration curve constructed at 245 nm using standard ethanolic solutions of atorvastatin. Freshly prepared Atorvastatin-NPs were subjected to ultracentrifugation (40000 rpm, 30 minutes), and the supernatant was collected to measure the concentration of unencapsulated (free) atorvastatin. The encapsulated drug content was obtained by subtracting the free drug amount from the total drug used in the formulation. DL and DEE values were calculated according to the following equations:

$$\mathrm{DL}\left(\%\right)=\frac{\mathrm{Total}\mathrm{ATOR}-\mathrm{free}\mathrm{ATOR}}{\mathrm{ATOR}-\mathrm{NPs}\mathrm{weight}}\times 100$$

$$\mathrm{DEE}\left(\%\right)=\frac{\mathrm{Total}\mathrm{ATOR}-\mathrm{free}\mathrm{ATOR}}{\mathrm{Total}\mathrm{ATOR}}\times 100$$

where Total ATOR represents the total amount of atorvastatin initially used in the formulation, Free ATOR represents the amount of nonencapsulated atorvastatin measured in the supernatant after ultracentrifugation, and ATOR-NPs weight represents the total weight of the recovered atorvastatin-loaded nanoparticles.

### 3.5. Animals

Thirty-six adult male laboratory C57BL/6 mice, aged 8 weeks and weighing 25 ± 3 g, were obtained from the animal facility of Kermanshah University of Medical Sciences. Animals were housed under controlled environmental conditions (temperature, 25 ± 2 °C; 12/12-hour light/dark cycle) with free access to chow and water. Animals were housed in separate cages according to their experimental groups throughout the study. All outcome analyses were performed at the individual-animal level.

The sample size (n = 6 animals per group) was selected based on previous studies employing the cuprizone-induced demyelination model and similar behavioral, histological, and immunohistochemical outcome measures. This sample size was considered sufficient to detect biologically meaningful differences while adhering to the ethical principle of minimizing animal use.

### 3.6. Induction of Cuprizone Demyelination and Experimental Groups

Demyelination was induced in all groups except the healthy control by incorporating 0.2% (w/w) cuprizone (CPZ) into the standard chow for 6 consecutive weeks, following the method described previously (38). Before group allocation, animals were weighed and distributed among groups to achieve comparable mean body weights at baseline. Animals were then assigned to 6 groups (n = 6 per group) (39-41) using coded identifiers, and treatment allocation remained concealed during drug administration.

Animals were grouped (n = 6 per group) as follows:

1) Healthy control: standard diet without CPZ.

2) CPZ group: diet containing 0.2% CPZ for 6 weeks.

3) Atorvastatin 1 mg/kg group: CPZ diet plus atorvastatin (1 mg/kg/day, i.p. route) during weeks 5 and 6.

4) Atorvastatin 2 mg/kg group: CPZ diet plus atorvastatin (2 mg/kg/day, i.p. route) during weeks 5 and 6.

5) Atorvastatin 4 mg/kg group: CPZ diet plus atorvastatin (4 mg/kg/day, i.p. route) during weeks 5 and 6.

6) Nano-atorvastatin 2 mg/kg group: CPZ diet plus nano-atorvastatin (2 mg/kg/day, i.p. route) during weeks 5 and 6.

All doses of atorvastatin were selected based on previous studies.

The primary outcome of the study was the extent of demyelination/remyelination in the corpus callosum, assessed by Luxol Fast Blue (LFB) staining. Secondary outcomes included behavioral performance (open field, pole test, and rotarod), NF-kB p65 immunoreactivity, and Nrf2 immunoreactivity.

### 3.7. Behavioral Tests

#### 3.7.1. Motor Coordination and Balance

Motor performance was evaluated at the end of the experimental period using the open-field, pole, and rotarod tests, as previously described with minor modifications (42-44). All behavioral assessments were performed in a quiet room under controlled lighting conditions by an investigator blinded to group allocation. Animals were identified only by coded labels throughout behavioral testing and data analysis.

Each mouse was acclimatized to the testing apparatus before data collection, and all measurements were performed by an investigator blinded to the treatment groups.

#### 3.7.2. Open-Field Test

The open-field apparatus consisted of a square arena measuring 40 × 40 cm with opaque walls 40 cm high. The floor was divided into 16 equal squares (10 × 10 cm). Each mouse was gently placed in the center of the arena, and its activity was recorded for 5 minutes. The total number of squares crossed (locomotor activity) was measured.

#### 3.7.3. Pole Test

Motor coordination and bradykinesia were assessed using a vertical pole 50 cm in height and 1 cm in diameter, wrapped with a rough-surfaced material to facilitate grip. Each mouse was placed head-upward near the top of the pole, and the time required to turn completely downward and descend to the base was recorded. Three trials were performed for each animal with 10-minute rest intervals, and the average time was calculated.

#### 3.7.4. Rotarod Test

The rotarod apparatus (Borj Sanat Azma ROTA-ROD M.T6700) was used to evaluate motor balance and coordination. Mice were placed on a rotating rod at a speed of 25 rpm over a 5-minute period. The latency to fall was recorded for each trial. Each animal underwent 3 trials with 10-minute rest breaks, and the mean latency was used for the study.

### 3.8. Tissue Preparation

On the final day of the experiment, after completion of behavioral testing, animals were deeply anesthetized with an intraperitoneal injection of ketamine (60 mg/kg) and xylazine (10 mg/kg). Mice were then euthanized, and the brains were quickly removed and fixed in 10% neutral-buffered formaldehyde for at least 72 hours. Fixed brains were processed for histological assays, including LFB staining of the corpus callosum (n = 3), as well as immunofluorescence analysis for the detection of Nrf2 and NF-kB p65 (n = 3).

### 3.9. Luxol Fast Blue Staining

The extent of demyelination in the corpus callosum was assessed using LFB staining. Paraffin-embedded sections (5 μm) were deparaffinized and hydrated to 95% ethanol and then incubated in 0.1% LFB solution overnight at 56 °C. Sections were differentiated in 0.05% lithium carbonate solution, followed by 70% ethanol, until the gray and white matter were clearly distinguishable. Slides were dehydrated, cleared in xylene, and mounted. LFB-stained images were analyzed using ImageJ software (NIH, USA). All images were processed using identical threshold settings. The positively stained myelinated area within the corpus callosum was quantified and expressed as the percentage of stained area relative to the total selected region of interest (33). Histological image acquisition and quantification were performed by an investigator blinded to treatment allocation using coded tissue samples.

### 3.10. Immunofluorescence Assay

Immunofluorescence was performed to detect the expression of NF-kB p65 and Nrf2 in the corpus callosum. Paraffin sections (5 μm) were deparaffinized, rehydrated, and subjected to antigen retrieval in citrate buffer (pH 7.4). After cooling, sections were washed with Tris-buffered saline (TBS) containing 0.03% Triton X-100 and blocked with 10% normal serum for 2 hours at room temperature. Slides were incubated overnight at 4 °C with primary antibodies (dilution, 1:100) specific for each marker, followed by 3 washes in TBS-T and incubation with an appropriate secondary antibody (dilution, 1:100) for 10 minutes at room temperature. Visualization was performed using DAPI. Finally, slides were dehydrated, cleared, and mounted for microscopic evaluation (45). Immunofluorescence image acquisition and quantitative analysis were performed under blinded conditions using coded specimens to minimize observer bias.

### 3.11. Statistical Analysis

The primary outcome of the study was the extent of demyelination in the corpus callosum, assessed by LFB staining. Secondary outcomes included behavioral performance in the open-field, pole, and rotarod tests, as well as immunofluorescence evaluation of NF-kB p65 and Nrf2 expression. Physicochemical characterization of the nanoformulation, including particle size, polydispersity index, zeta potential, drug loading, encapsulation efficiency, and in vitro drug release, was also performed.

Statistical analyses were performed using GraphPad Prism version 9 (San Diego, CA, USA). One-way ANOVA and two-way ANOVA (for assessing body-weight variation across the 6-week period), followed by the Tukey post hoc test, were applied to evaluate group differences. Results are presented as mean ± SEM, and all estimates are presented with 95% confidence intervals (95% CI). Values of P < 0.05 were considered statistically significant. Before ANOVA, data normality was assessed using the Shapiro-Wilk test. The results confirmed that the data were normally distributed (P > 0.05), supporting the use of parametric statistical analyses. Quantitative evaluation of histological sections was performed using ImageJ software (Windows platform). For statistical analyses, each animal was considered an independent experimental unit. When multiple images or tissue sections were obtained from the same animal, quantitative measurements were averaged to generate a single value per animal before group-level statistical analysis.

## 4. Results

### 4.1. Physicochemical Characterization

#### 4.1.1. Particle Size Distribution

Dynamic light scattering analysis showed that atorvastatin-loaded nanoparticles had a Z-average diameter of 100.5 nm with a PDI of 0.392, indicating a relatively narrow size distribution. The intensity-based distribution curve revealed 3 peaks: a major population at 136.8 nm (84.7%), a minor peak at 33.5 nm (12.3%), and a trace population at 5148 nm (3.1%). These results suggest that the formulation predominantly consisted of nanoparticles in the desirable sub-200-nm range, with acceptable polydispersity for biological applications.

#### 4.1.2. Zeta Potential

The surface charge of atorvastatin nanoparticles was determined by electrophoretic light scattering. The average zeta potential was -43.6 mV, with 2 major peaks at -37.9 mV (70.8%) and -58.4 mV (29.2%). This high negative charge indicates good colloidal stability and suggests strong electrostatic repulsion among particles, thereby preventing aggregation and maintaining dispersion stability.

#### 4.1.3. Drug Loading and Entrapment Efficiency

The optimized formulation demonstrated a DL of approximately 20% w/w and a DEE of approximately 86%, values consistent with lipid-based nanoparticles designed for highly lipophilic drugs. The high encapsulation efficiency may be attributed to the crystalline-imperfect lipid matrix, which provides sufficient accommodation sites for atorvastatin molecules.

#### 4.1.4. In Vitro Drug Release

**Figure 1.**
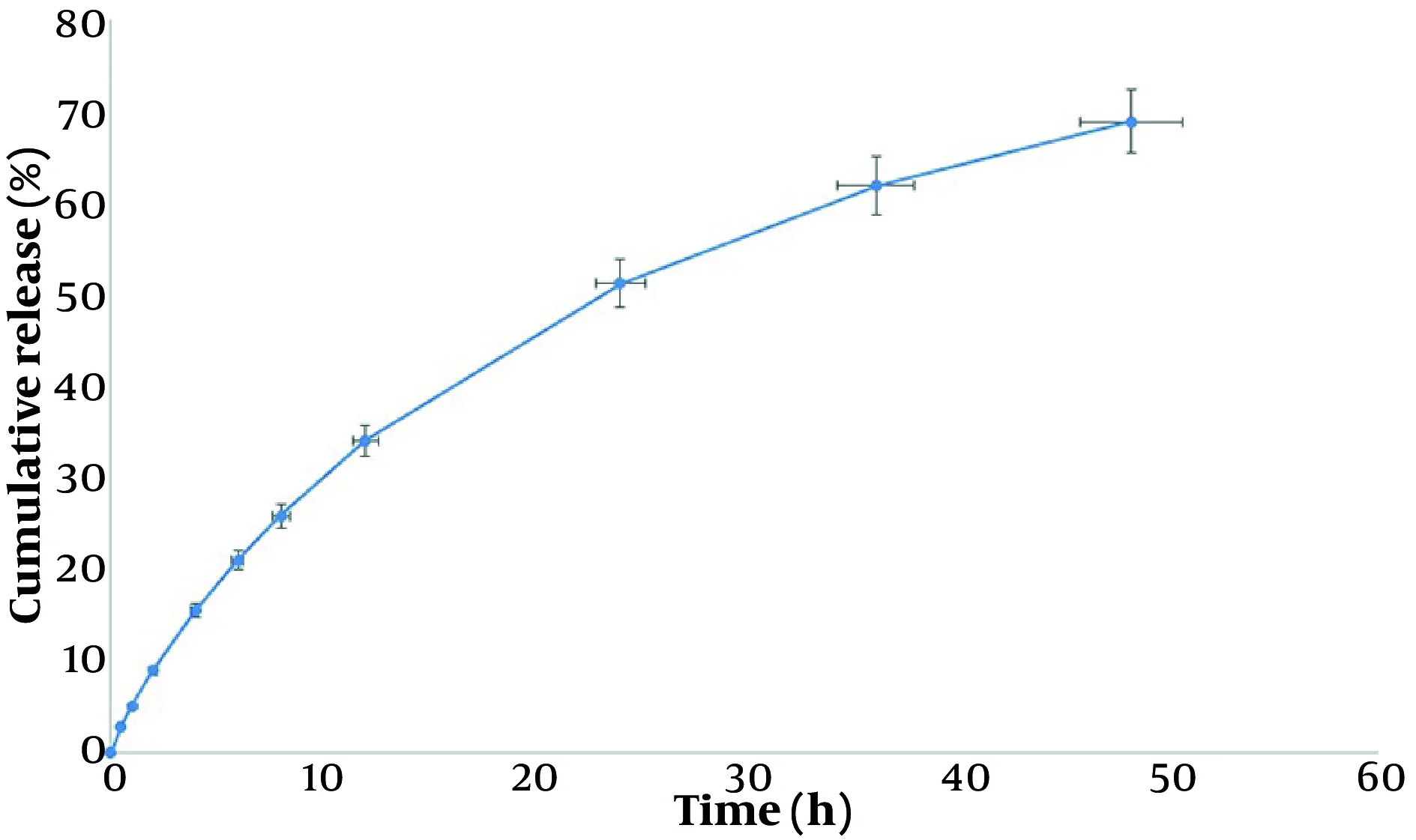
Release percentage of atorvastatin from nano-atorvastatin.

The release study demonstrated a sustained-release profile, with approximately 68% of atorvastatin released over 48 hours in PBS (pH 7.4, 37 °C) under sink conditions. The release kinetics fitted well to diffusion-based models, such as the Higuchi/Weibull models, consistent with drug diffusion through a solid lipid matrix as the predominant mechanism. This sustained pattern contrasts with the rapid release of free drug, highlighting the ability of NPs to prolong atorvastatin delivery (Figure 1).

#### 4.1.5. Fourier Transform Infrared Spectroscopy Analysis

**Figure 2.**
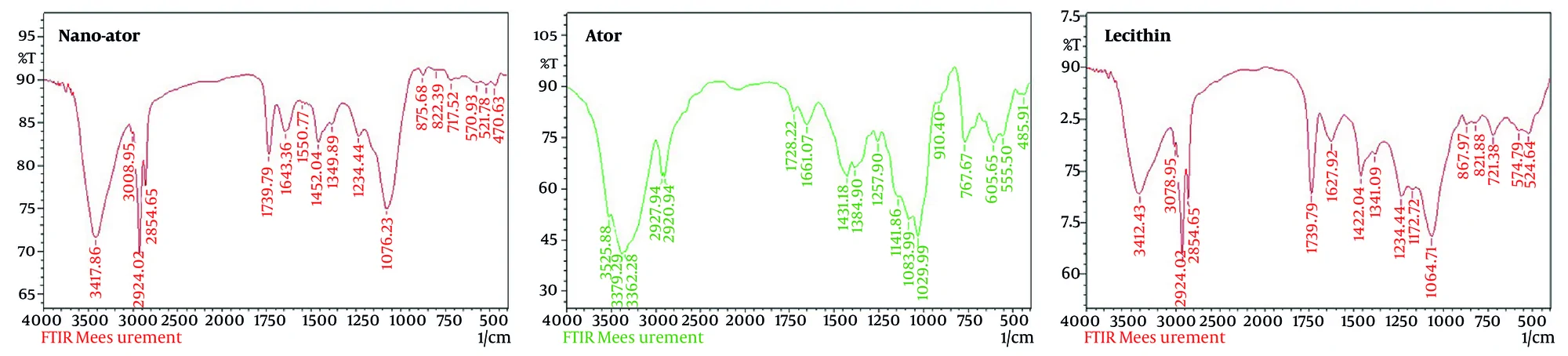
FT-IR spectra of Ator (atorvastatin), Nano-Ator (nano-atorvastatin), and lecithin.

Atorvastatin FTIR spectra (Figure 2) showed a broad absorption at 3368 cm^-1^, indicating hydrogen bonding and O-H/N-H stretching vibrations. At 2923 and 2853 cm^-1^, prominent aliphatic C-H stretching bands were observed. Two distinct carbonyl absorptions were identified: A conjugated carbonyl/amide vibration at 1651 cm^-1^ and a strong peak at 1723 cm cm^-1^, which was attributed to a free carbonyl group (ester or carboxyl). C-O and C-N stretching modes were associated with bands at 1276 to 1009 cm^-1^, whereas aromatic C=C stretching was observed in the 1600 to 1500 cm^-1^ region. The presence of hydroxyl, carbonyl, aromatic, and aliphatic groups was further supported by out-of-plane aromatic C-H deformations at 910 and 768 cm^-1^ (46, 47).

The broad band at 3320 to 3310 cm^-1^ in the lecithin FTIR spectra was indicative of O-H/N-H stretching vibrations. At 2920 and 2850 cm^-1^, strong aliphatic C-H stretching absorptions were detected. Ester carbonyl (C=O) stretching was identified by a prominent peak at 1735 cm^-1^, whereas C=C stretching of unsaturated fatty acids was indicated by bands at 1650 to 1620 cm^-1^. Out-of-plane =C-H bending was observed at 810 to 720 cm^-1^, phosphate deformation bands were observed at approximately 520 to 570 cm^-1^, and phosphate-related absorptions were observed at 1240 to 1170 cm^-1^ and 1060 to 970 cm^-1^. These characteristics confirm the presence of long-chain fatty acid, phosphate, carbonyl, and hydroxyl groups (48, 49). The major bands of lecithin and atorvastatin remained present in the FTIR spectra of atorvastatin-lecithin nanoparticles, but discernible shifts and variations in intensity were observed. Enhanced hydrogen bonding was suggested by the broad O-H/N-H band at 3320 cm^-1^, which was wider than in the individual spectra. Aliphatic C-H stretching remained noticeable at 2920 and 2850 cm^-1^. Although the lecithin carbonyl peak at 1735 cm^-1^ remained intact, the atorvastatin carbonyl signal at 1650 cm^-1^ showed a modest shift and decreased intensity, indicating drug-lipid interactions. With only minor changes, phosphate vibrations were maintained between 1240 and 1170 cm^-1^ and between 1060 and 970 cm^-1^. Overall, the observed spectral shifts and intensity variations indicate nanoparticle formation and confirm the effective integration of atorvastatin into the liposomal matrix.

#### 4.1.6. UV Assay

**Figure 3.**
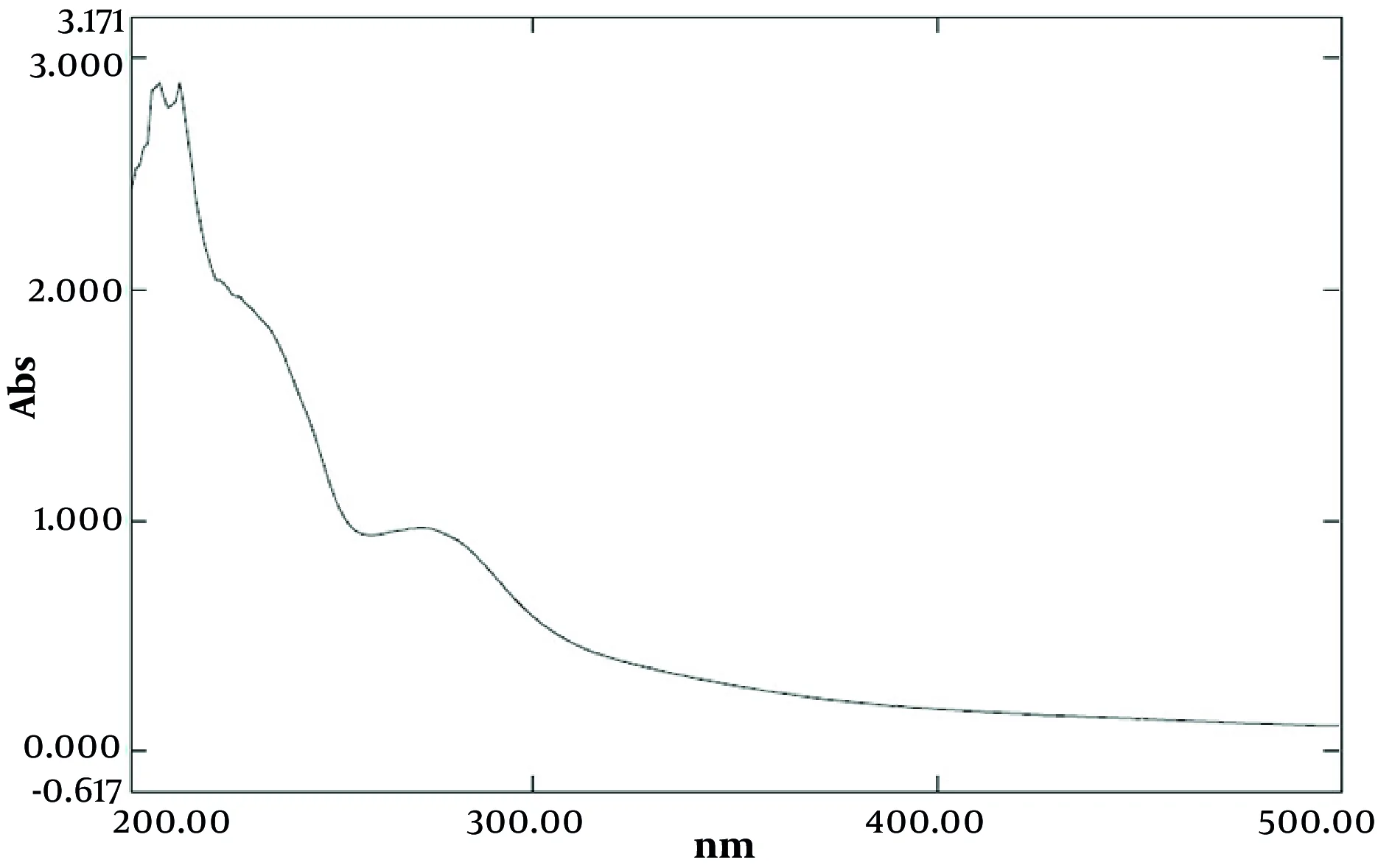
UV absorption of nano-atorvastatin.

Figure 3 indicates that nano-atorvastatin was synthesized and that its absorption was at 245 nm.

#### 4.1.7. Stability Note

No visible aggregation was observed during the release study. Post-assay measurements confirmed that particle size and zeta potential remained within the same range as those of freshly prepared samples, supporting the structural stability of the system.

### 4.2. Animal Study

All animals remained healthy throughout the experimental period. No deaths, exclusions, or losses occurred during the study. Therefore, all 36 mice completed the experimental protocol and were included in the behavioral, histological, and immunofluorescence analyses (n = 6 per group for all outcomes).

#### 4.2.1. Body Weight Changes

**Figure 4.**
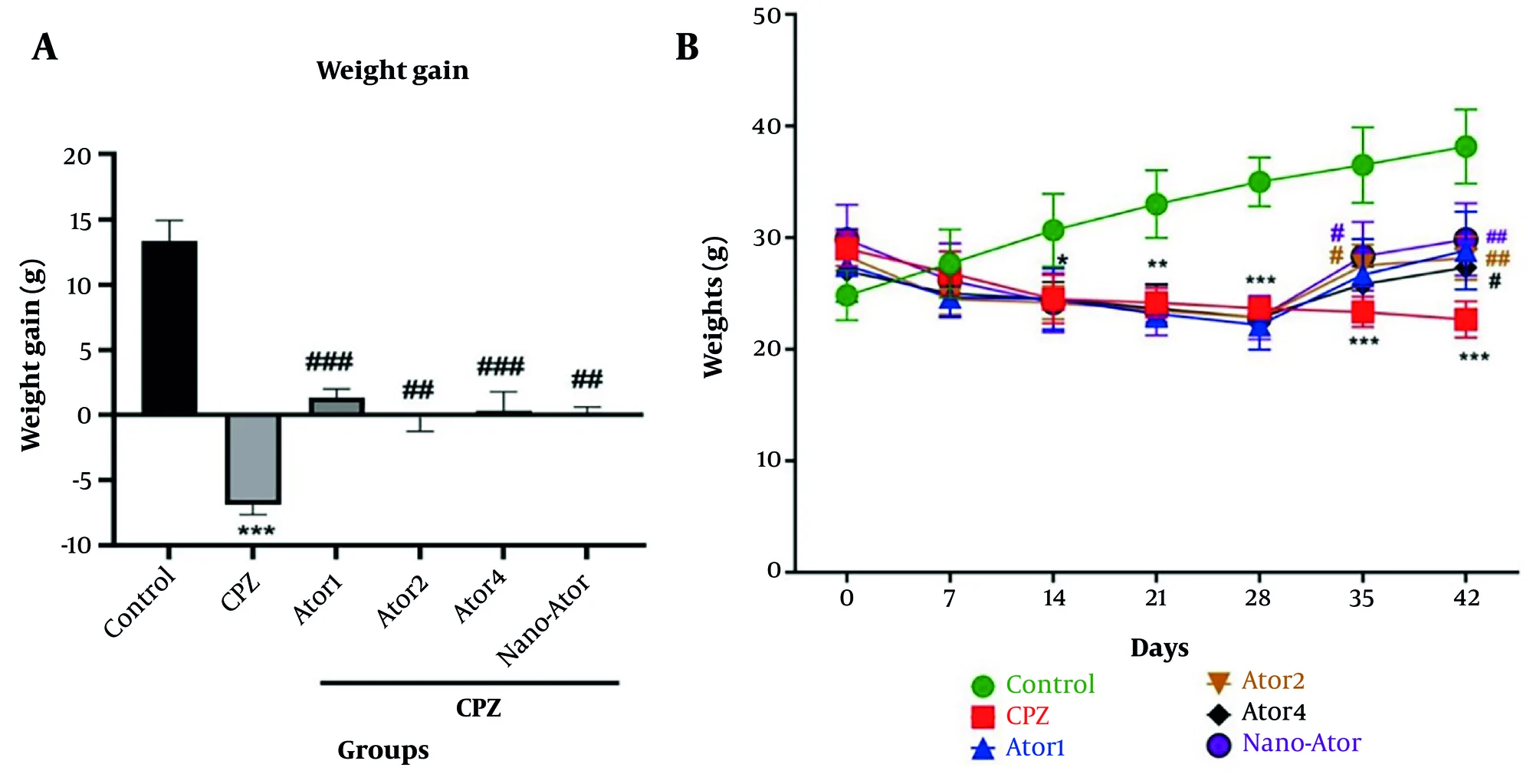
Effects of CPZ, atorvastatin (1, 2, and 4 mg/kg), and nano-atorvastatin (2 mg/kg) on body weight; A, Weight gain during the experimental period; B, Weekly changes in body weight. Data are presented as mean ± SEM (n = 6). ***P < 0.001 vs Control; #P < 0.05, ##P < 0.01, ###P < 0.001 vs CPZ.

Body weight analysis revealed that CPZ administration caused a significant reduction in weight gain compared with the control group (F (5, 30) = 35.35, P < 0.001). Treatment with atorvastatin at different doses, as well as nano-atorvastatin, partially prevented this loss. As shown in Figure 4A, CPZ markedly reduced weight gain, whereas atorvastatin at 1 mg/kg (P = 0.0002), 2 mg/kg (P = 0.0023), and 4 mg/kg (P = 0.001), and the nano-formulation (P < 0.01), attenuated this decline. Weekly follow-up of body weight (Figure 4B) confirmed this finding, showing a continuous increase in the control group, a progressive decrease in CPZ-treated animals (F (30, 180) = 21.81, P < 0.001), and significant improvement in the treatment groups.

#### 4.2.2. Open-Field Test

**Figure 5.**
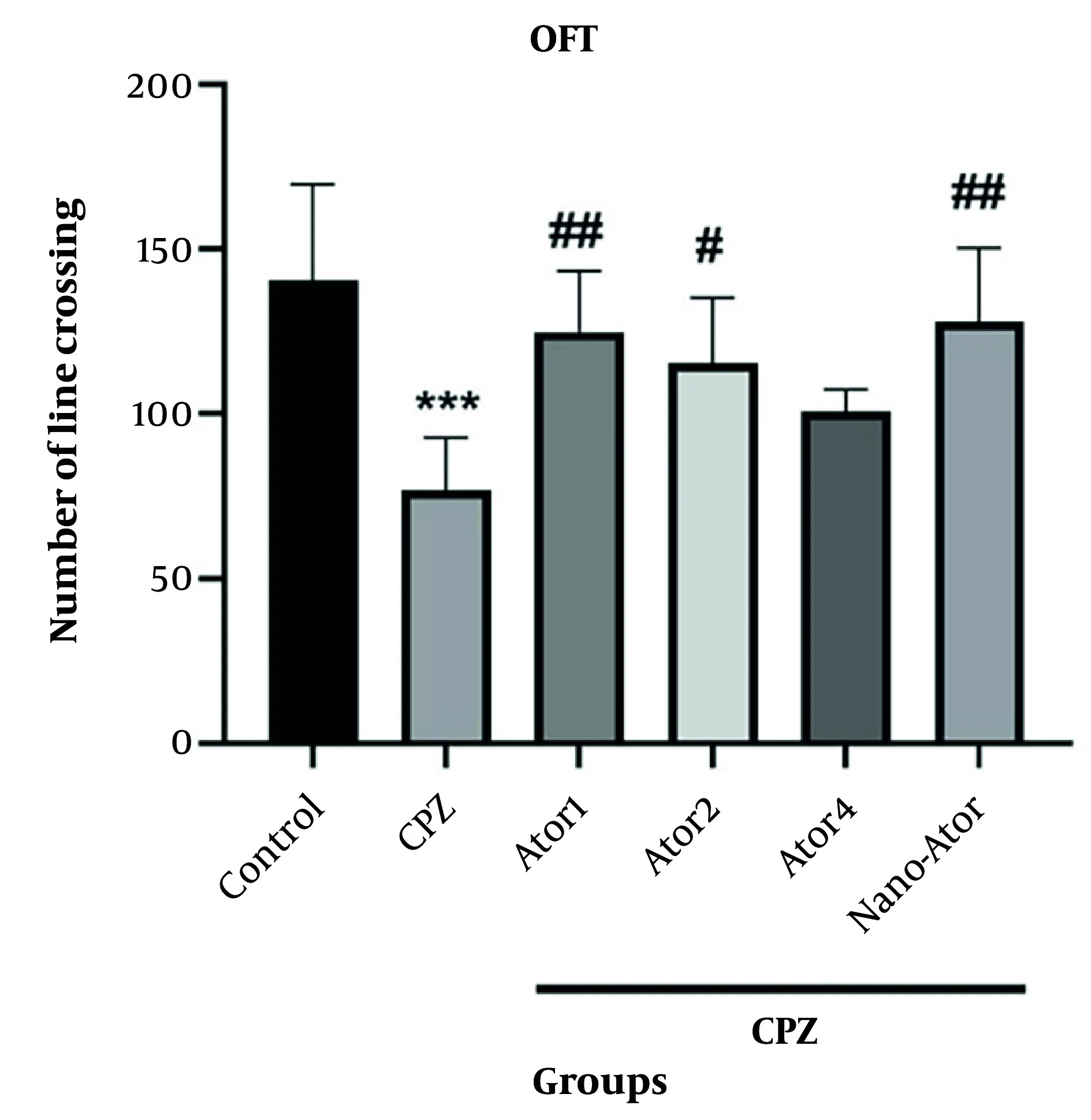
Effect of CPZ and treatments on locomotor activity in the open-field test. Data are presented as mean ± SEM (n = 6). ***P < 0.001 vs Control; #P < 0.05, ##P < 0.01 vs CPZ.

In the open-field test (Figure 5), the number of line crossings was markedly reduced in the CPZ group (F (5, 27) = 7.623, P = 0.0001), indicating impaired locomotor activity. Treatment with atorvastatin at 1 and 2 mg/kg (P = 0.0057 and P = 0.0377, respectively) and nano-atorvastatin (P = 0.0029) significantly increased locomotor activity compared with the CPZ group. These results demonstrate the protective effects of atorvastatin in reversing CPZ-induced behavioral deficits.

#### 4.2.3. Pole Test

**Figure 6.**
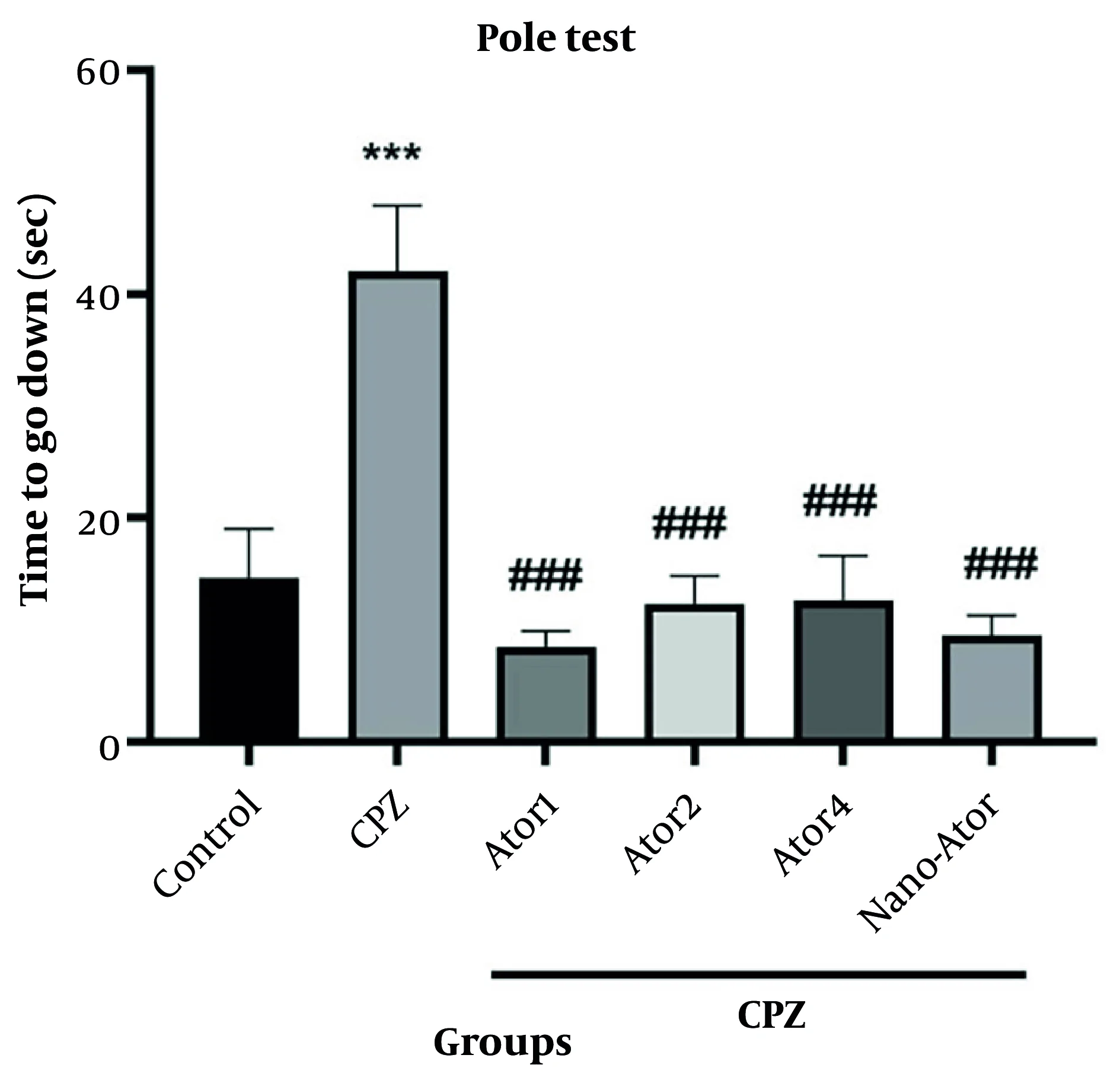
Effect of CPZ and treatments on motor coordination in the pole test. Data are presented as mean ± SEM (n = 6). ***P < 0.001 vs Control; ###P < 0.001 vs CPZ.

The time required to descend the pole was significantly prolonged in the CPZ group compared with the control group (F (5, 30) = 11.87, P < 0.0001) (Figure 6), reflecting impaired motor coordination. Atorvastatin at all tested doses, as well as nano-atorvastatin, significantly reduced the descent time (P < 0.0001), restoring performance close to the control level.

#### 4.2.4. Rotarod Test

**Figure 7.**
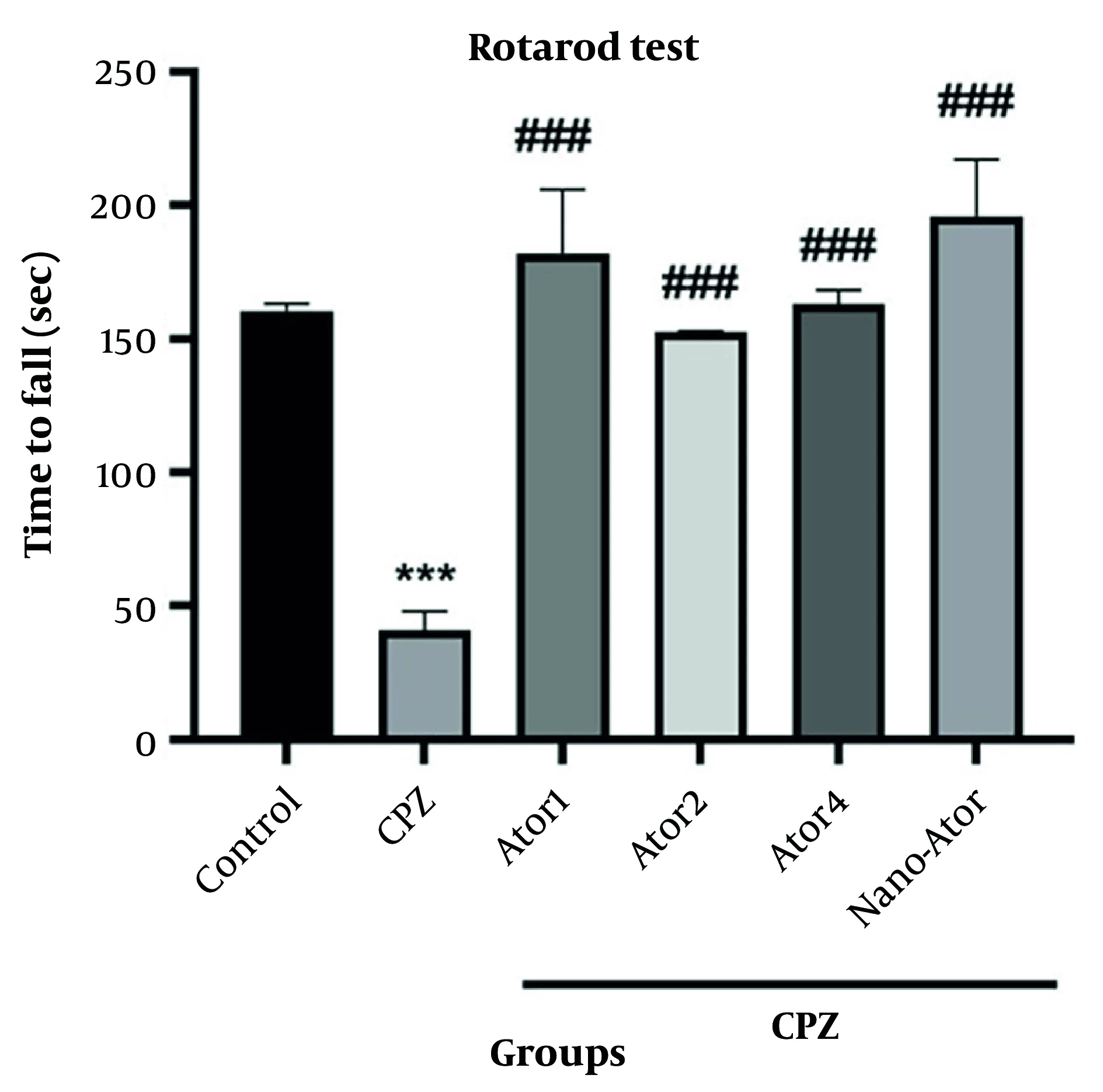
Effect of CPZ and treatments on motor balance in the rotarod test. Data are presented as mean ± SEM (n = 6). ***P < 0.001 vs Control; ###P < 0.001 vs CPZ.

The latency to fall in the rotarod test was markedly decreased in CPZ-treated mice (F (5, 30) = 16.58, P < 0.0001), indicating severe motor impairment. Treatment with atorvastatin and nano-atorvastatin significantly increased the time spent on the rod (P < 0.0001), suggesting a strong protective effect against CPZ-induced motor dysfunction (Figure 7).

### 4.3. Histological and Immunohistochemical Analysis

#### 4.3.1. LFB Staining

**Figure 8.**
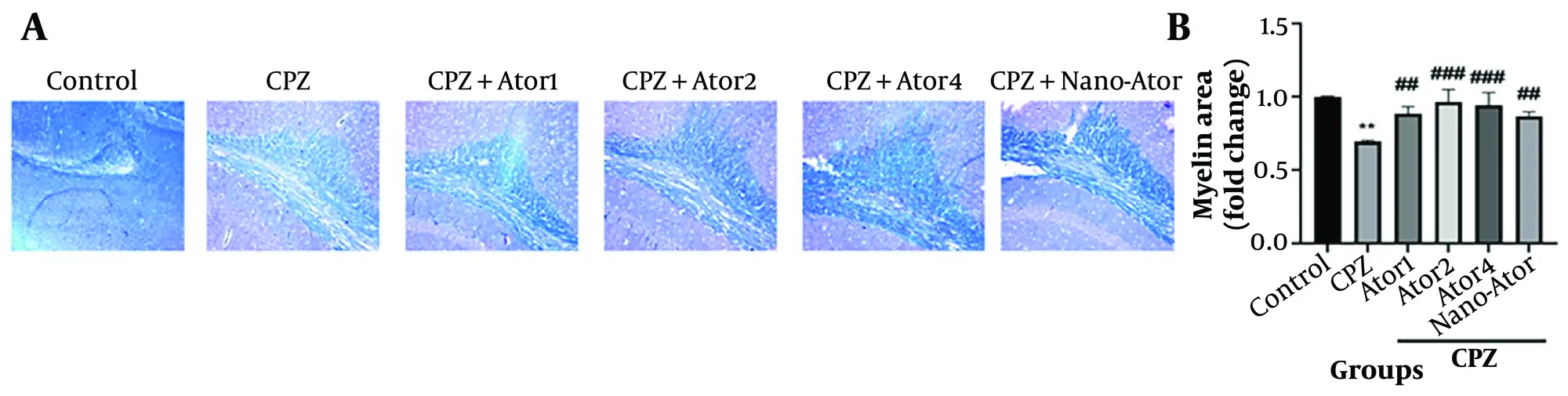
Luxol Fast Blue (LFB) staining of the corpus callosum. (A) Representative photomicrographs from control, CPZ, and CPZ + atorvastatin (1, 2, or 4 mg/kg) or nano-atorvastatin groups. (B) Quantification of myelin area (fold change) analyzed with ImageJ. Data are expressed as mean ± SEM. *P < 0.01 vs control; ##P < 0.01, ###P < 0.001 vs CPZ.

One-way ANOVA showed a significant difference among the experimental groups (F (5, 18) = 15.46, P < 0.0001). As shown in Figure 8, control mice displayed intense blue staining in the corpus callosum, indicating intact myelin. In contrast, the CPZ group showed a marked reduction in myelin density, reflecting extensive demyelination compared with the control group (P < 0.0001). Treatment with atorvastatin at doses of 1 mg/kg (P = 0.0015), 2 mg/kg, and 4 mg/kg (P < 0.0001), as well as nano-atorvastatin (P = 0.0040), significantly ameliorated CPZ-induced myelin loss, as indicated by increased LFB intensity and myelin area quantification compared with the CPZ group.

#### 4.3.2. NF-kB p65 Immunoreactivity

**Figure 9.**
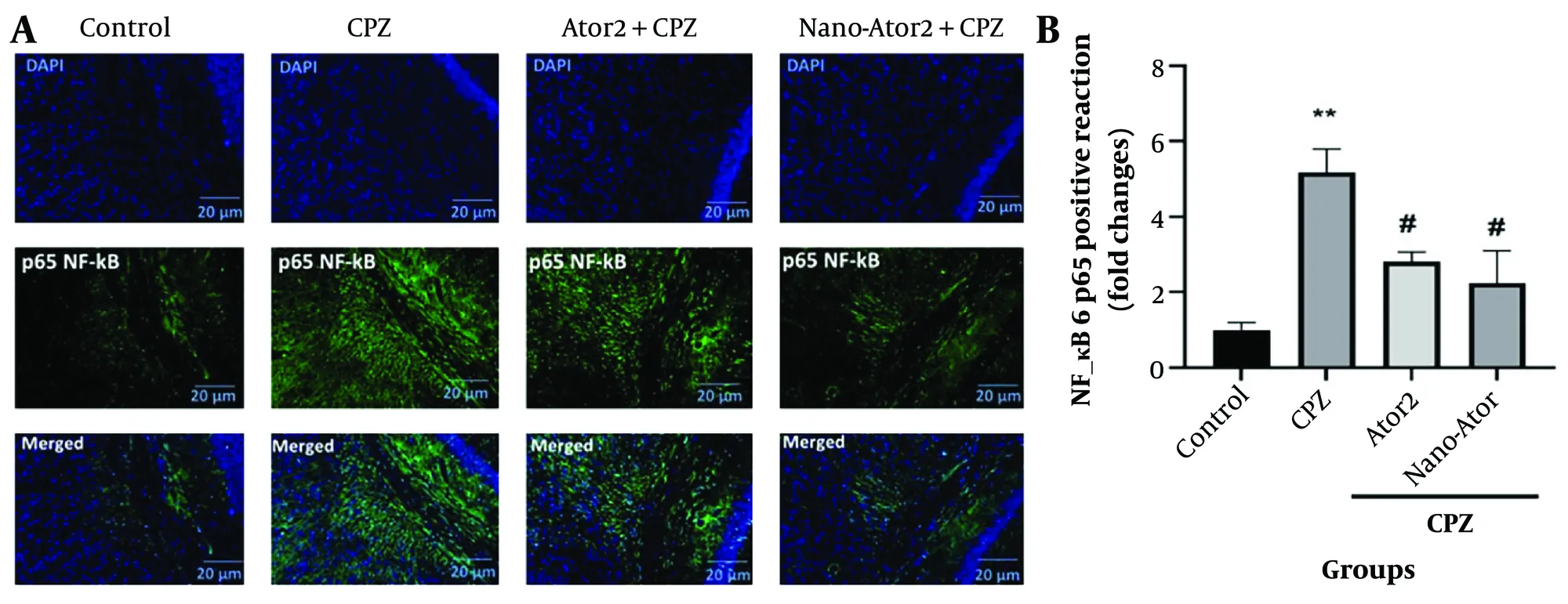
NF-kB p65 immunofluorescence in the corpus callosum; A. Representative DAPI (blue), NF-kB p65 (green), and merged images for control, CPZ, atorvastatin (2 mg/kg), and nano-atorvastatin groups; B, Quantitative analysis of NF-kB p65-positive cells. Data are mean ± SEM. *P < 0.01 vs control; #P < 0.05 vs CPZ.

One-way ANOVA showed a significant difference among the experimental groups (F (3, 4) = 19.92, P = 0.0072). Immunofluorescence staining revealed low NF-kB expression in control mice, whereas CPZ exposure markedly increased p65 NF-kB nuclear positivity (P = 0.0058 vs the control group) (Figure 9). Administration of atorvastatin (2 mg/kg) (P = 0.0430) or nano-atorvastatin (P = 0.0206) substantially reduced NF-kB activation in the corpus callosum compared with the CPZ group, supporting the anti-inflammatory role of atorvastatin, with the nano-formulation showing comparable efficacy.

#### 4.3.3. p-Nrf2 Immunoreactivity

**Figure 10.**
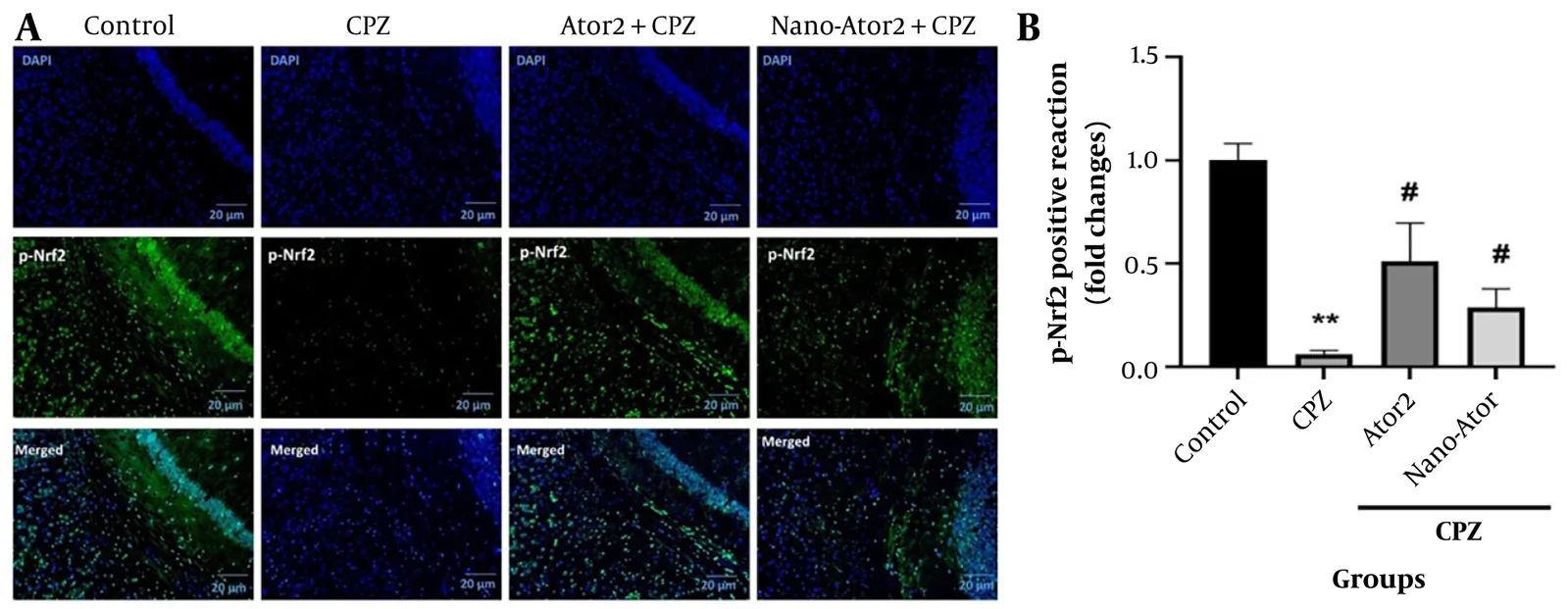
Phospho-Nrf2 immunofluorescence in the corpus callosum; A. Representative DAPI (blue), p-Nrf2 (green), and merged images for control, CPZ, atorvastatin (2 mg/kg), and nano-atorvastatin groups; B, Quantification of p-Nrf2 nuclear-positive cells (fold change). Data are mean ± SEM. *P < 0.01 vs control; #P < 0.05 vs CPZ.

To assess the antioxidant pathway, phosphorylated p-Nrf2 was examined (Figure 10). One-way ANOVA showed a significant difference among the experimental groups (F (3, 4) = 25.89, P = 0.0044). Control mice exhibited robust nuclear localization of p-Nrf2, whereas CPZ intoxication significantly decreased its expression (P = 0.0038). Both atorvastatin 2 mg/kg and nano-atorvastatin partially restored p-Nrf2 immunoreactivity in the corpus callosum (P = 0.0394 and P = 0.0106 vs the CPZ group, respectively), although levels did not reach those of controls. These results highlight the modulatory effect of atorvastatin on the NF-kB/Nrf2 axis in demyelination.

## 5. Discussion

The present study investigated the neuroprotective potential of atorvastatin and its nano-formulation in the CPZ model of demyelination. The CPZ model is among the most widely used experimental paradigms for investigating MS pathology, primarily because of its reproducibility and the selective injury it induces in oligodendrocytes (50). In our study, 6 weeks of CPZ feeding induced marked weight loss, motor impairment, and histopathological alterations, confirming the robustness of the model and enabling evaluation of the therapeutic effects of atorvastatin. The key findings demonstrate that atorvastatin, and particularly its nano-formulation, ameliorates behavioral dysfunction, preserves myelin integrity, and modulates molecular signaling pathways related to inflammation and oxidative stress (51). These findings are significant in the context of MS research, in which inflammation, demyelination, and neurodegeneration coexist and drive disease progression.

Behavioral tests are crucial for linking molecular and histological findings to functional outcomes. CPZ exposure led to profound deficits in locomotor coordination and balance, as reflected in open-field, pole, and rotarod performance. These impairments are consistent with previous studies reporting that CPZ-induced demyelination disrupts neuronal conduction and motor behavior (53). Treatment with atorvastatin significantly improved motor coordination, suggesting preservation of axonal integrity and myelin. Atorvastatin treatment showed significant improvement compared with the CPZ group, with no significant difference between the nano and conventional forms; this finding is consistent with the hypothesis that enhanced bioavailability and brain penetration augment therapeutic efficacy. These behavioral improvements not only support the neuroprotective potential of atorvastatin but also suggest that nano-formulation could provide an alternative approach for CNS drug delivery, warranting further investigation.

Histopathological evaluation revealed profound cortical abnormalities in CPZ-treated mice, including vacuolation, disrupted cortical architecture, and hypercellularity. These findings reflect neuronal loss, glial activation, and structural disorganization typically observed in demyelinating models. LFB staining demonstrated significant myelin preservation in the atorvastatin groups. Quantitative analysis confirmed increased myelin density compared with CPZ alone, supporting the role of atorvastatin in protecting or restoring myelin (50). Collectively, these immunohistochemical findings provide strong evidence that atorvastatin counters CPZ-induced demyelination.

NF-kB plays a central role in neuroinflammatory cascades. Its activation has been implicated in MS pathology and in CPZ-induced demyelination, where it drives the expression of proinflammatory cytokines, nitric oxide synthase, and adhesion molecules. In our study, CPZ exposure markedly increased NF-kB p65 nuclear positivity, reflecting heightened inflammatory responses (52). Atorvastatin treatment suppressed this activation, consistent with its known anti-inflammatory effects. Similar effects were observed following treatment with nano-atorvastatin, with both formulations reducing NF-kB p65 immunoreactivity compared with the CPZ group. These findings align with previous research showing that statins inhibit NF-kB signaling in various neuroinflammatory conditions, thereby reducing cytokine production and improving neuronal survival. By attenuating NF-kB, atorvastatin may reduce astrocytosis and microglial activation, both of which are key drivers of demyelination.

In parallel with inflammation, oxidative stress is a major contributor to demyelination. CPZ toxicity involves ROS generation, mitochondrial dysfunction, and subsequent oligodendrocyte apoptosis. Nrf2, a transcription factor that orchestrates antioxidant defenses, is downregulated in CPZ models, leading to impaired cellular resilience. In the present study, CPZ markedly reduced Nrf2 immunoreactivity in the corpus callosum, whereas conventional atorvastatin and the nano-formulation similarly restored its expression (51, 53). This restoration implies enhanced transcription of antioxidant genes such as HO-1 and NQO1, thereby countering oxidative stress and promoting cell survival. Activation of Nrf2 has been reported as a protective mechanism in several demyelinating and neurodegenerative models. Our results therefore support the hypothesis that atorvastatin exerts neuroprotection not only through anti-inflammatory mechanisms but also by strengthening antioxidant responses.

The concurrent suppression of NF-kB and activation of Nrf2 is particularly noteworthy. These pathways often act in opposition: NF-kB amplifies inflammation, whereas Nrf2 promotes cytoprotection. An imbalance between the 2 contributes to progressive neuronal injury in MS. By restoring this balance, atorvastatin may create an environment conducive to remyelination and functional recovery. Modulation of both NF-kB and Nrf2 signaling by atorvastatin and nano-atorvastatin may represent an important mechanism underlying the behavioral and histological improvements observed following treatment.

Several therapeutic agents have been evaluated in the CPZ model, including antioxidants such as resveratrol (54) and quercetin (55), anti-inflammatory agents such as dapsone (56) and metformin (57), and natural compounds including rutin (38). Most of these interventions exert protective effects through modulation of oxidative stress and inflammatory responses. The beneficial effects observed with atorvastatin in the present study are generally consistent with these mechanisms and support the potential of statins as neuroprotective agents in demyelinating disorders. In addition, atorvastatin offers the advantage of extensive clinical experience and a well-established safety profile in cardiovascular medicine, which may facilitate future translational development (58).

Previous clinical studies investigating statins in patients with MS have yielded variable results, with some reports demonstrating beneficial effects on inflammatory activity and disease progression, whereas others have shown limited efficacy (58, 59). These discrepancies may reflect differences in study design, statin type, dosage regimen, treatment duration, and patient characteristics. In the present study, both conventional atorvastatin and nano-atorvastatin improved behavioral, histopathological, and molecular outcomes compared with the CPZ group. Although the nano-formulation did not consistently demonstrate superiority over conventional atorvastatin, these findings suggest that formulation-related factors may influence therapeutic performance and warrant further investigation.

Mechanistically, the observed protective effects were associated with modulation of both NF-kB and Nrf2 signaling pathways. Excessive NF-kB activation contributes to neuroinflammation and tissue injury, whereas impaired Nrf2 signaling weakens endogenous antioxidant defenses and may exacerbate demyelination. Therefore, interventions capable of regulating both pathways may provide a multifaceted therapeutic approach by simultaneously reducing inflammation and enhancing cellular protection. The present findings indicate that both atorvastatin and nano-atorvastatin modulate the NF-kB/Nrf2 axis and may contribute to the behavioral and histopathological improvements observed in the cuprizone model.

Finally, given atorvastatin's long history of safe use in cardiovascular medicine, repurposing it for MS may accelerate its path toward clinical translation. Large, well-controlled clinical trials assessing nano-formulations are warranted to determine whether the benefits observed in the CPZ model translate to human disease.

### 5.1. Study Limitations

This study has several limitations that should be considered when interpreting the findings. First, the study was conducted exclusively in male mice; therefore, potential sex-dependent differences in treatment response were not evaluated. Second, although the sample size was consistent with previous studies using the cuprizone model, larger cohorts may provide greater statistical power to detect subtle differences between conventional and nano-formulated atorvastatin. Third, the treatment period was relatively short and does not fully reflect the chronic nature of MS. Fourth, although the nano-formulation demonstrated favorable physicochemical characteristics and biological activity, direct pharmacokinetic measurements and brain drug-distribution analyses were not performed. Finally, the cuprizone model reproduces important features of demyelination but does not fully recapitulate the complex immune and clinical manifestations of human MS. Therefore, further studies involving long-term treatment protocols, both sexes, pharmacokinetic evaluation, and additional preclinical models are warranted to confirm the translational potential of these findings.

### 5.2. Conclusions

In summary, this study demonstrated that atorvastatin and its nano-formulation exert neuroprotective effects in the cuprizone model of demyelination. Treatment improved behavioral performance, preserved myelin integrity, and modulated key molecular pathways by suppressing NF-kB-mediated inflammation while enhancing Nrf2-dependent antioxidant defenses. The nano-formulation exhibited superior efficacy compared with conventional atorvastatin, highlighting the importance of optimized drug delivery systems for CNS disorders. These findings suggest that atorvastatin, especially in nano-form, holds promise as a therapeutic candidate for demyelinating diseases such as MS. Further studies, including dose-response evaluations, mechanistic analyses, and clinical trials, are necessary to validate and translate these results.

## Data Availability

The dataset presented in the study is available on request from the corresponding author during submission or after publication.
